# Microrna-217 modulates human skin fibroblast senescence by directly targeting DNA methyltransferase 1

**DOI:** 10.18632/oncotarget.16509

**Published:** 2017-03-23

**Authors:** Ben Wang, Rui Du, Xiao Xiao, Zhi-Li Deng, Dan Jian, Hong-Fu Xie, Ji Li

**Affiliations:** ^1^ Department of Dermatology, Xiangya Hospital, Central South University, Changsha, China; ^2^ Department of Dermatology, Hunan Provincial People's Hospital, Changsha, China; ^3^ Key Laboratory of Organ Injury, Aging and Regenerative Medicine of Hunan Province, Changsha, China; ^4^ Center for Molecular Medicine, Xiangya Hospital, Central South University, Changsha, China

**Keywords:** DNMT1, miR-217, senescence, skin aging

## Abstract

DNA methyltransferase 1 (DNMT1) is a major epigenetic regulator associated with many biological processes. However, the roles and mechanisms of DNMT1 in skin aging are incompletely understood. Here we explored the role of DNMT1 in human skin fibroblasts senescence and its related regulatory mechanisms. DNMT1 expression decreased in passage-aged fibroblasts and DNMT1 silencing in young fibroblasts induced the senescence phenotype. MiR-217 is predicted to target DNMT1 mRNA and miR-217 expression increased in passage-aged fibroblasts. MiR-217 directly targeted the 3′-untranslated region (3′-UTR) of DNMT1 in HEK 293T cells and inhibited DNMT1 expression in fibroblasts. MiR-217 overexpression induced a senescence phenotype in young fibroblasts, and miR-217 downregulation in old HSFs partially reversed the senescence phenotype. However, these effects could be significantly rescued by regulating DNMT1 expression in fibroblasts. After regulating miR-217 levels, we analyzed changes in the promoter methylation levels of 24 senescent-associated genes, finding that 6 genes were significantly altered, and verified p16 and phosphorylated retinoblastoma (pRb) protein levels. Finally, an inverse correlation between DNMT1 and miR-217 expression was observed in skin tissues and different-aged fibroblasts. Together, these findings revealed that miR-217 promotes fibroblasts senescence by suppressing DNMT1-mediated methylation of p16 and pRb by targeting the DNMT1 3′-UTR.

## INTRODUCTION

Aging is a phenomenon that is associated with a gradual decline in biological functions. Cellular senescence of human skin fibroblasts (HSFs) has been proposed to be major underlying cause of skin aging [1]. Cellular senescence is not only regulated by genetic factors but also by epigenetic mechanisms, including DNA methylation, histone modification, chromatin remodeling, and non-coding RNA regulation [2].

DNA methyltransferase 1 (DNMT1), the major enzyme responsible for maintaining DNA methylation patterns, is located at the replication fork and methylates newly biosynthesized DNA to induce gene silencing [3, 4]. DNMT1 functions have been studied extensively. It has been shown to play important roles in tumor development [5], heart diseases [6], and autoimmune diseases [7], among other biological processes. Furthermore, the expression of DNMT1 decreased gradually in both premature and replicative senescent human embryonic lung fibroblasts [7], and DNMT1 knockdown caused telomere shortening in glioma cell lines [8]. Several studies have demonstrated a correlation between DNMT1 and the classical senescence-related gene p16 in some cancer cells [9–11].

In addition, we previously showed the involvement of DNMT1 in some premature-senescence phenomena, including uneven epidermal thickness, shorter and thinner hair fibers in K14-Cre-mediated DNMT1 knockout mice [12]. Therefore, we speculated that DNMT1 might play a key role in skin aging. However, the roles and mechanisms of DNMT1 in skin aging have not been reported so far.

As mentioned above, DNMT1 can regulate the expression of important genes involved in many different biological processes. Recent data have revealed that the Wnt/β-catenin signaling pathway can contribute to the stability of DNMT1 expression in urological cancer cell lines [13]. Lin et al. [14] reviewed that Ras-c-Jun signaling pathway can induce transcriptional up-regulation of DNMT1. In contrast, p53 and FOXO3a can transcriptionally repress DNMT1 expression. During the post-translational modification of DNMT1 proteins, ubiquitin-like proteins containing plant homeodomains and class I histone deacetylases are known to control the stability of DNMT1 [15, 16], and AKT, PKCζ, and CDKs 1, 2, and 5 can control phosphorylation-mediated DNMT1 stability [14]. In addition, microRNA (miRNA)-mediated regulation of DNMT1 expression has also been reported. Previously, it was shown that miR-185 and GKN1-miR-185-DNMT1 axis can suppress breast carcinoma proliferation and inhibit hepatocellular carcinoma growth [17, 18]. MiR-152, modulated activation of the canonical Wnt pathway by targeting DNMT1 [19] and could form a feedback loop with DNMT1 in Nis-transformed cells [20]. MiR-148b and miR-126 were also shown to be associated with DNMT1 expression [21, 22]. Most of these studies related to DNMT1 expression were performed using cancer cells. At present, miRNAs that directly target DNMT1 expression have not been reported for skin cell senescence.

Though bioinformatics analysis, we found that miR-217 potentially targets DNMT1 mRNA. miR-217 consists of 23 nucleotides, is located in Chromosome 2, and has been demonstrated to function as a potential tumor suppressor in several types of carcinoma cells by targeting KRAS, WASF3, and Sirt1 mRNAs [23, 24]. MiR-217 was identified as an endogenous inhibitors of Sirt1, which promoted endothelial cell senescence [25]. To date, there were not further study demonstrating an association between miR-217 and cellular senescence.

Here we studied the role of DNMT1 and its regulatory mechanisms in HSFs senescence. We found that DNMT1 knockdown could induce HSF senescence and that miR-217 can target the 3′-UTR sequence of DNMT1 and induce senescence in HSFs. Moreover, the effect of miR-217 in promoting senescence was associated with decreased p16 and phosphorylated retinoblastoma (pRb) gene methylation levels, resulting from DNMT1 downregulation. Finally, we demonstrated an inverse correlation between DNMT1 and miR-217 expression in skin tissues and HSFs from patients of different ages, implying that miR-217 and DNMT1 contribute to the pathogenesis of skin aging.

## RESULTS

### DNMT1 expression decreased in passage-aged HSFs and its silencing induced HSF senescence

**Figure 1:**
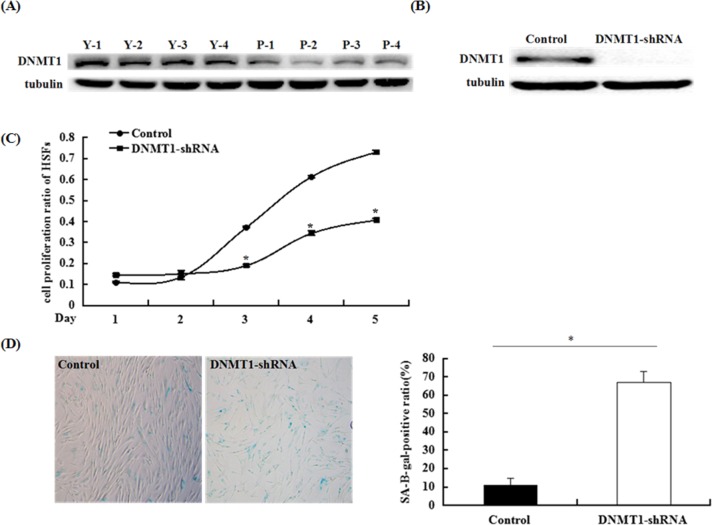
DNMT1 expression decreased in passage-aged HSFs, and DNMT1 silencing induced HSF senescence **(A)** DNMT1 levels were detected by western blot analysis. DNMT1 expression in young HSFs was significantly higher than that observed in passage-aged HSFs. (n = 10, *p < 0.05). DNMT1 expression levels of 4 representative couples are shown (Y: young HSFs; P: passage-aged HSFs). **(B)** DNMT1 levels were detected by western blot analysis. DNMT1 expression in HSFs was significantly silenced using a lentivirus expressing an shRNA against DNMT1 after 48 h (*p < 0.05). **(C)** The HSF growth rate was determined by performing MTT assays. The growth rate of HSFs transduced with the DNMT1-shRNA lentivirus was significantly decreased compared with HSFs transduced with the control shRNA lentivirus. (n = 3 for each time point, *p < 0.05). **(D)** HSF senescence was evaluated by measuring the number of SA-β-gal-positive cells (left panel). The SA-β-gal-positive rate was obviously enhanced in HSFs transduced with the DNMT1-shRNA lentivirus, compared with that observed in control transductants (right panel). (n = 3, *p < 0.05)

As mentioned above, DNMT1 may be closely associated with skin aging. Thus, we initially measured DNMT1 expression during the passaging of HSFs into senescence. Primary HSFs were isolated from normal skin tissues, which were gathered from the UV-unexposed areas of young patients undergoing plastic surgery, and passaged *in vitro*. We evaluated DNMT1 expression in 10 patient-matched pairs of HSFs that had undergone different numbers of passages by western blot analysis, finding that DNMT1 protein expression in passage-aged HSFs was markedly lower in HSFs from patients in the younger cohort (Figure 1A).

To explore the role of DNMT1 in cellular senescence, we transduced young HSFs with a lentivirus vector expressing an shRNA against DNMT1 or a control lentivirus. Subsequently, we studied senescence-associated indicators, including senescence-associated β-galactosidase (SA-β-gal) activity and the proliferation rate. By western blot analysis, we confirmed that the DNMT1 shRNA efficiently decreased DNMT1 protein expression, relative to that observed in the control-shRNA group (Figure 1B). Silencing DNMT1 expression caused a significant increase of SA-β-gal-positive cells and decreased cell proliferation at 48 h post-transfection (Figure 1C, 1D). These data demonstrated that DNMT1 knockdown induced an erupted senescent phenotype in HSFs and that DNMT1 could play an important role in skin aging.

### Regulation of DNMT1 expression by miR-217 in HSFs

**Figure 2:**
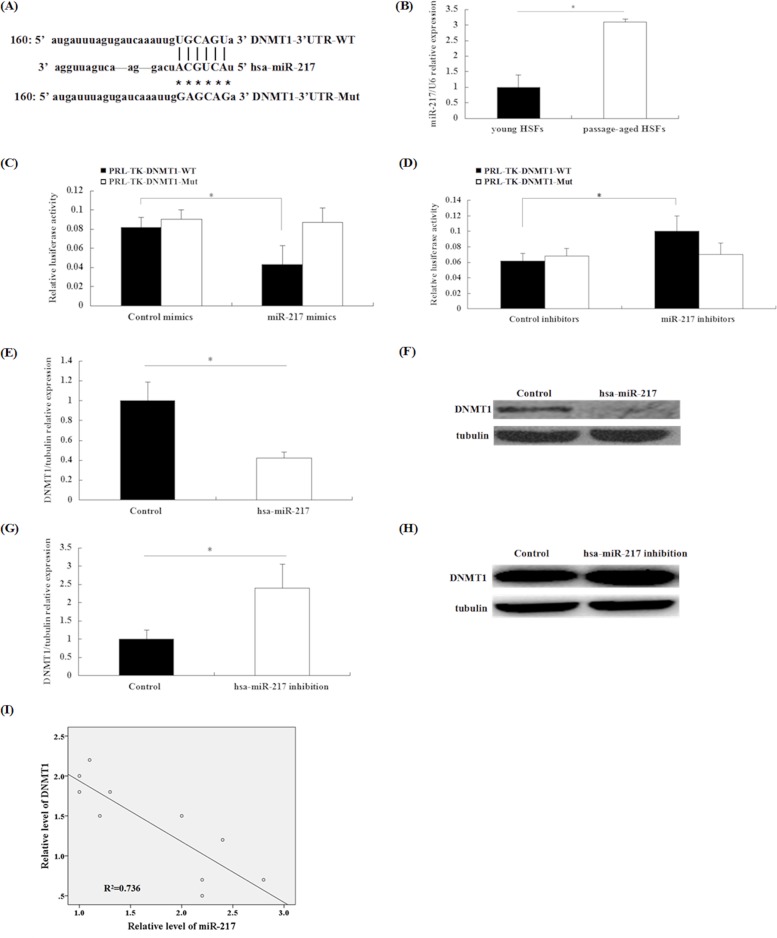
DNMT1 is a target of miR-217 and regulation of DNMT1 expression by miR-217 in HSFs **(A)** Bioinformatics analysis was used to predict an miR-217-binding site in the 3′-UTR of DNMT1, as shown in the upper site. Mutated residues are shown in the lower site. **(B)** miR-217 expression was detected by RT-qPCR. miR-217 expression was significantly upregulated in passage-aged HSFs, compared with that observed in young HSFs (n = 3, *p < 0.05). **(C)** Luciferase activity of the WT luciferase reporter vector, but not the Mut 3′-UTR reporter, decreased in 293T cells transfected with an miR-217 mimics (n = 3, *p < 0.05). **(D)** Luciferase activity of the WT reporter vector, but not the Mut 3′-UTR reporter, increased in 293T cells transfected with an miR-217 inhibitors (n = 3, *p < 0.05). **(E, F)** DNMT1 mRNA and protein levels were detected by RT-qPCR and western blotting, respectively. DNMT1 levels were significantly downregulated after transducing HSFs with a lentivirus expressing hsa-miR-217 (n = 3, *p < 0.05). **(G, H)** DNMT1 mRNA and protein levels were detected by RT-qPCR and western blot, respectively. DNMT1 levels were upregulated after transducing HSFs with a lentivirus expressing an hsa-miR-217 inhibitors (n = 3, *p < 0.05). **(I)** miR-217 levels were negatively correlated with DNMT1 protein levels in different couples of young and passage-aged HSFs (n = 10, R^2^ = 0.736, *p < 0.05).

While searching for an upstream miRNA that could down-modulate DNMT1 expression, we found homology between miR-217 and a region in the 3′-UTR of human DNMT1 RNA, based on computational miRNA target analysis with miRNA databases (www.microrna.org; Figure 2A). Next, we studied the miR-217 level in senescent HSFs and found that miR-217 was significantly upregulated in passage-aged HSFs, compared with those from the younger cohort (Figure 2B). To determine whether miR-217 directly targeted DNMT1, we generated a WT DNMT1 3′UTR luciferase reporter vector and a mutant (Mut) DNMT1 3′-UTR luciferase reporter vector. We cotransfected HEK293T cells with an miR-217 mimics or a control mimics along with either reporter vector. Compared with the control mimics, the miR-217 mimics caused a 40% reduction in luciferase activity in samples cotransfected with WT reporter vector, while no significant variation was observed in the samples cotransfected with the Mut reporter vector. Similar results were observed in HSFs transfected with an miR-217 inhibitors versus a control inhibitors (Figure 2C, 2D). These results confirmed that miR-217 could directly target the 3′-UTR of DNMT1.

As DNMT1 was identified as an miR-217 target, we next sought to determine whether miR-217 could surpress DNMT1 expression in HSFs. We found that DNMT1 expression was significantly downregulated after transduction with an hsa-miR-217 lentivirus in young HSFs, while DNMT1 levels increased in old HSFs transduced with a lentivirus expressing an hsa-miR-217 inhibitors, as assessed by reverse-transcriptase quantitative PCR (RT-qPCR) and western blot analysis (Figure 2E–2H and Supplementary Figure 1).

Moreover, we analyzed the correlation between miR-217 and DNMT1 levels in 10 couples of HSFs with differing passage numbers. The results showed an inverse correlation of the DNMT1 protein and miR-217 expression in HSFs passaged different numbers of times in culture (R^2^ = 0.736, P < 0.05; Figure 2I).

### Regulation of cellular senescence in HSFs by miR-217

**Figure 3:**
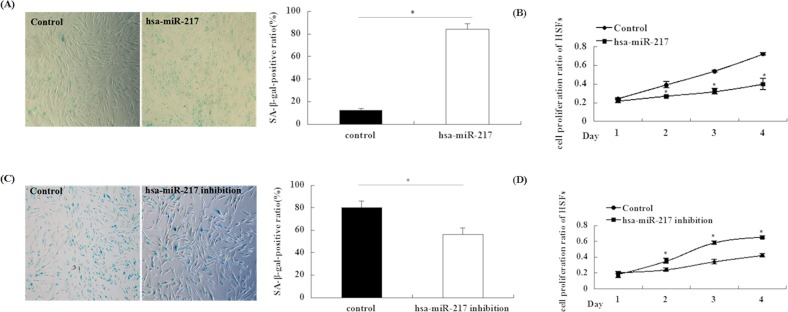
miR-217 could induce senescence in HSFs **(A)** HSFs senescence was detected by evaluating the number of SA-β-gal positive cells at 48 h post-transduction with a lentivirus expressing hsa-miR-217 in young HSFs (left panel). The SA-β-gal-positive rate was obviously enhanced in HSFs transduced with the hsa-miR-217 lentivirus, compared with the negative-control lentivirus (right panel). (n = 3, *p < 0.05). **(B)** The HSF growth rate was determined by performing MTT assays. The cellular growth rate significantly decreased in young HSFs transduced with the hsa-miR-217 lentivirus compared with the control lentivirus (n = 3 for each time point, *p < 0.05). **(C)** HSF senescence was evaluated by measuring the percentage of SA-β-gal-positive cells at 48 h post-transduction with a lentivirus expressing an hsa-miR-217 inhibitors in passage-aged HSFs (left panel). The SA-β-gal-positive rate was obviously decreased in HSFs transduced with the hsa-miR-217 inhibitors lentivirus, compared with the control lentivirus (right panel). (n = 3, *p < 0.05). **(D)** The HSF growth rate was measured by performing MTT assays. The cellular growth rate was significantly increased in passage-aged HSFs transduced with the hsa-miR-217 inhibitors lentivirus compared with the control lentivirus (n = 3 for each time point, *p < 0.05).

To determine the role of miR-217 in HSF senescence, we examined senescence-related indexes after modulating the miR-217 level. In young HSFs, miR-217 overexpression increased the percentage of SA-β-gal-positive cells and decreased the cell proliferation rate, compared with that observed in the control group (Figure 3A, 3B). Conversely, in passage-aged HSFs, miR-217 inhibition significantly decreased the percentage of SA-β-gal-positive cells and increased the proliferation rate, compared with the control group (Figure 3C, 3D). Thus, our results indicated that miR-217 could improve senescence in HSFs.

### MiR-217 promotes senescence by suppressing DNMT1 expression in HSFs

**Figure 4:**
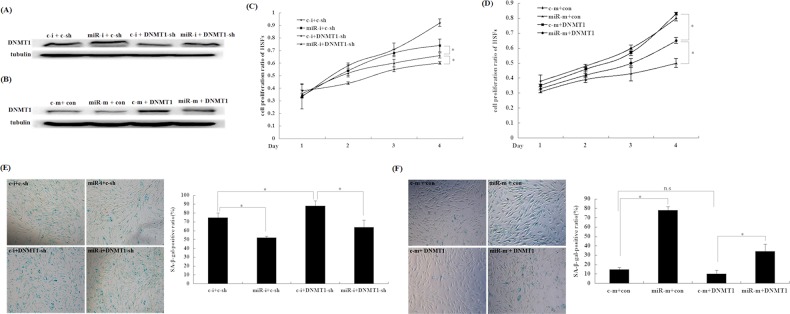
miR-217 suppresses DNMT1 expression during HSF senescence Abbreviations: c-i: control inhibitors; miR-i: miR-217 inhibitors; c-sh: control shRNA; DNMT1-sh: DNMT1 shRNA lentivirus; c-m: control mimics; miR-m: miR-217 mimics; con: control adenovirus; DNMT1: DNMT1 adenovirus. **(A)** DNMT1 expression was detected by western blot analysis. The DNMT1 shRNA lentivirus partially reversed DNMT1 upregulation caused by the miR-217 inhibitors (*p < 0.05). **(B)** DNMT1 expression was detected by western blotting. The DNMT1 adenovirus partially reversed DNMT1 downregulation caused by the miR-217 mimics (*p < 0.05). **(C)** HSF growth rates were determined by performing MTT assays. The DNMT1-shRNA lentivirus partially reversed the increased proliferation rate caused by the miR-217 inhibitors (n = 3 for each time point, *p < 0.05). **(D)** HSF growth rates were detected by performing MTT assays. The DNMT1 adenovirus could partially reverse the decreased proliferation rate caused by the miR-217 mimics (n = 3 at each time point, *p < 0.05). **(E)** HSF senescence was measured by determining the percentage of SA-β-gal-positive cells. Transduction with the DNMT1 shRNA lentivirus partially reversed the decreased SA-β-gal-positive ratio caused by the miR-217 inhibitors (*p < 0.05; left panel). Quantification of the SA-β-gal-positive rate is shown in the right panel (n = 3, *p < 0.05). **(F)** HSF senescence was evaluated by determining the number of SA-β-gal-positive cells. The DNMT1 adenovirus did significantly inhibit the increased SA-β-gal-positive ratio caused by the miR-217 mimics (*p < 0.05; left panel). Quantification of the SA-β-gal-positive ratio is shown in the right panel (n = 3, *p < 0.05).

To examine whether miR-217 induces senescence in HSFs through DNMT1, we increased or silenced DNMT1 expression in HSFs with overexpressed or down-modulated miR-217 (Figure 4A, 4B). We found that DNMT1 could partially reverse both the miR-217-mediated increase in SA-β-gal activity and decreased proliferation rate in young HSFs (Figure 4C, 4D). Conversely, silencing DNMT1 partially reversed the decreased SA-β-gal activity and increased proliferation rate caused by miR-217 inhibition in passage-aged HSFs (Figure 4E, 4F). Collectively, these findings indicated that miR-217 promoted HSF senescence by suppressing DNMT1 expression.

### Role of miR-217 in modulating promoter methylation levels of senescent-associated genes in HSFs

**Figure 5:**
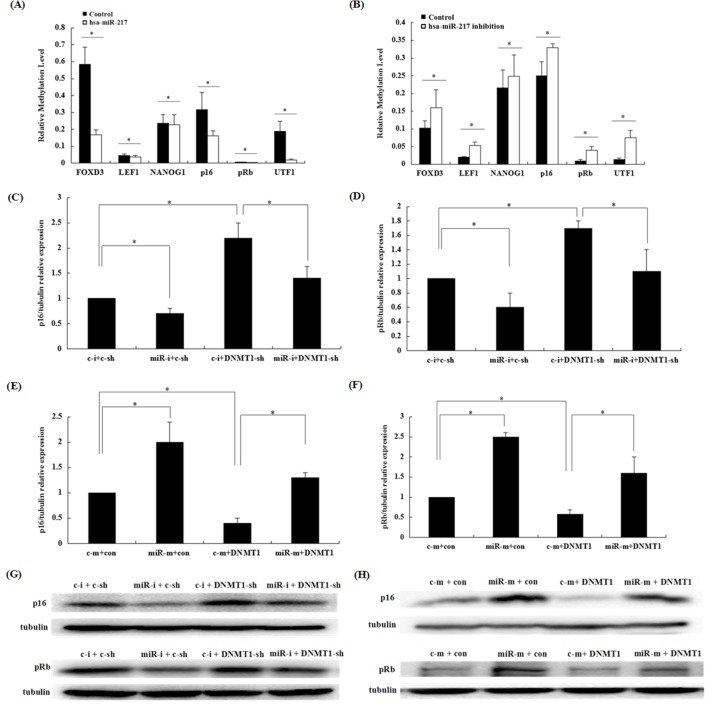
Role of miR-217 in modulating the promoter-methylation levels of p16 and pRb in HSFs Abbreviations: c-i: control inhibitors; miR-i: miR-217 inhibitors; c-sh: control shRNA; DNMT1-sh: DNMT1 shRNA lentivirus; c-m: control mimics; miR-m: miR-217 mimics; con: control adenovirus; DNMT1: DNMT1 adenovirus. **(A)** After HSFs were transduced with the hsa-miR-217 lentivirus, the promoter-methylation levels of 24 senescence-associated genes were detected using microfluidic PCR and next-generation bisulfite sequencing. The relative methylation levels of 6 affected genes are shown (*p < 0.05). **(B)** After HSFs were transfected with the hsa-miR-217 inhibitors lentivirus, the promoter-methylation levels of 24 senescence-associated genes were detected using microfluidic PCR and next-generation bisulfite sequencing. The relative methylation levels of 6 affected genes are shown (*p < 0.05). **(C, D)** p16 and pRb mRNA expression levels were detected by RT-qPCR. The DNMT1 shRNA lentivirus partially reversed the decreased p16 or pRb expression levels caused by the miR-217 inhibitors (*p < 0.05). **(E, F)** p16 or pRb mRNA expression was detected by RT-qPCR. The DNMT1 adenovirus partially reversed the enhanced p16 or pRb expression caused by the miR-217 mimics (*p < 0.05). **(G)** p16 or pRb protein expression was detected by western blotting. The DNMT1 shRNA lentivirus partially reversed the decreased p16 or pRb expression caused by the miR-217 inhibitors (*p < 0.05). **(H)** p16 or pRb protein expression was detected by western blotting. The DNMT1 adenovirus partially reversed the heightened p16 or pRb expression caused by the miR-217 mimics (*p < 0.05).

To determine whether miR-217-dependent DNMT1 down-regulation could affect DNA methylation levels of the promoters of senescent-associated genes, we studied the effects of miR-217 expression on DNA methylation in the promoters of 24 senescent-associated genes, using microfluidic PCR and next-generation bisulfite sequencing (Supplementary Table 1). Only 6 detected genes had significantly alteration, and DNA-methylation analysis showed that miR-217 variation altered the promoter methylation levels of 6 senescence-associated genes (Figure 5A, 5B). Among these 6 senescence-associated genes, expression of the classical senescence biomarker p16 and its downstream target pRb were further confirmed by RT-PCR and western blotting. The p16 and pRb mRNA and protein levels increased after miR-217 overexpression and decreased after miR-217 inhibition, compared with their expression levels in control cells. Moreover, the changes in p16 and pRb expression levels mediated by miR-217 were partially reversed by regulating DNMT1 expression (Figure 5C–5H). These data suggested that miR-217 induced a hypomethylation-dependent increase in p16 and pRb expression, likely due to its effect on DNMT1 translation, leading to HSF senescence.

### miR-217 and DNMT1 expression in skin tissues and HSFs collected from individuals of different ages

**Figure 6:**
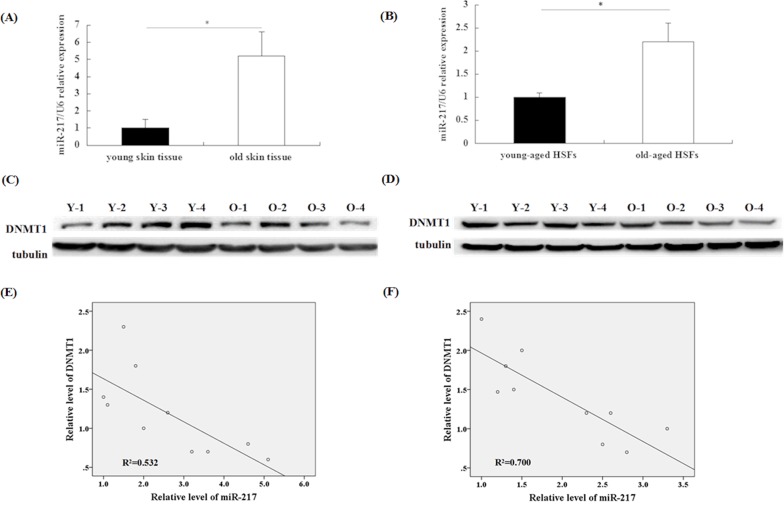
miR-217 and DNMT1 expression *in vivo* **(A)** miR-217 levels were detected by RT-qPCR. miR-217 expression was significantly upregulated in old skin tissues compared with that observed in young skin tissues (n = 10, *p < 0.05). **(B)** miR-217 levels were detected by RT-qPCR. miR-217 expression was significantly upregulated in old-aged HSFs compared with that observed in young-aged HSFs (n = 10, *p < 0.05). **(C)** DNMT1 expression was detected by western blotting. DNMT1 expression in old skin tissues was significantly lower than that observed in young skin tissues (n = 10, *p < 0.05). DNMT1 expression in 4 representative couples is shown (Y: young skin tissues; O: old skin tissues). **(D)** DNMT1 expression was detected by western blotting. DNMT1 expression in old-aged HSFs was significantly repressed compared with that measured in young-aged HSFs (n = 10, *p < 0.05). DNMT1 expression of 4 representative couples is shown (Y: young-aged HSFs; O: old-aged HSFs). **(E)** miR-217 levels were negatively correlated with DNMT1 protein levels in different couples of young and old skin tissues (n = 10, R^2^ = 0.532, *p < 0.05). **(F)** miR-217 levels were negatively correlated with DNMT1 protein levels in different couples of young-aged and old-aged HSFs (n = 10, R^2^ = 0.700, *p < 0.05).

Functional changes in senescent HSFs *in vivo* may play an important role in skin aging. Therefore, to investigate the expression of miR-217 and DNMT1 *in vivo*, we examined their expression levels in normal skin tissues and HSFs from skin tissues of patients of different ages who had undergone plastic surgery. The results confirmed that miR-217 expression was significantly upregulated in skin tissues and HSFs from old individuals (Figure 6A, 6B). DNMT1 protein expression in skin tissues or HSFs from old individuals were remarkably lower than that in the younger cohort (Figure 6C, 6D). In contrast, a negative correlation was observed between miR-217 and DNMT1 levels in skin tissues (R^2^ = 0.532, P < 0.05) and HSFs (R^2^ = 0.700, P < 0.05) from individuals of different ages (Figure 6E, 6F).

## DISCUSSION

Aging is a process of slow and gradual deterioration of functional capacities, which makes individuals more prone to a variety of illnesses and leads to a dramatic reduction of the probability of survival. Skin aging is the most visible sign of aging across organisms and is induced by genetic factors, stochastic events, the environment, and other non-genetic factors. Recently, epigenetics has become recognized as an important contributor to the aging process [26]. Epigenetics refers to phenotypic or gene-expression differences that do not involve changes in the underlying DNA sequence, which may result from DNA methylation, histone modification, chromatin remodeling, and non-coding RNA regulation [27].

DNA methylation is catalyzed by DNMTs, and DNMT1 is the most abundant DNMT involved in preserving and propagating existing methylation patterns during cell division. Lin SP et al. reported that the Rb gene could maintain quiescence and prevent premature senescence through DNMT1 upregulation in mesenchymal stromal cells [28], implying a possible role for DNMT1 in aging. We demonstrated for the first time that DNMT1 can also protect HSFs from senescence in this study. Previous epigenetic studies have provided evidence that DNMT1 can influence p16 signaling [29], PTEN/Akt signaling [30], and sirt1 recruitment [31], mechanisms that also may be used by DNMT1 in regulating HSF senescence. As described above, DNMT1 can be directly targeted or indirectly influenced by several miRNAs, which raises the possibility that some miRNAs could target DNMT1 during senescence in HSFs. Though bioinformatics analysis and dual-luciferase reporter assays, we confirmed that DNMT1 could be targeted by miR-217. This finding represents the first demonstration that DNMT1 is a target of miR-217. This finding is in agreement with previous data that miR-217 induces senescence in endothelial cells [25]. Taken together, our findings revealed that miR-217 improves senescence in HSFs by targeting DNMT1.

As mentioned above, miR-217 can influence Sirt1 expression, and Sirt1 inhibition can decrease p53 acetylation [32] and induce the expression of senescent-associated secretory phenotype (SASP) [33], indicating that miR-217 might induce senescence via a Sirt1 or SASP-mediated pathway. Dachshund homolog 1, another known target gene of miR-217, can repress cyclin D1 expression to influence the cell cycle [34]. PTEN can also be targeted by miR-217 to dysregulate PI3K/Akt/PTEN signaling-induced, oxidative-associated senescence [35, 36], implying miR-217 may influence HSF senescence by regulating the cell cycle or the PI3K/Akt/PTEN pathway. This study provides the first demonstration that miR-217 can induce senescence by targeting DNMT1 in relation to skin aging. DNMT1 has been associated with many tumor types and correlates with cell growth, apoptosis, aging, and death [37, 38]. However, the mechanisms whereby miR-217 induces HSF senescence by targeting DNMT1 have not been investigated.

Results from previous studies have revealed that DNMT1 can increase DNA methylation of the PTEN promoter, influence AKT phosphorylation, regulate the p16INK4a-cyclin D1-CDK 4/6-pRb-E2F1 pathway and pRb/E2F1 pathway [39, 40], and repress p53 levels to maintain cell survival [41]. These findings imply that DNMT1 inhibition might induce senescence though several well-studied senescence pathways. Based on the targeting relationship between miR-217 and DNMT1, we assessed the promoter-methylation levels of 24 senescence-associated genes using microfluidic PCR and next-generation bisulfite sequencing, finding 6 senescence-associated genes with altered promoter-methylation levels in HSFs. The classical senescence markers (p16 and pRb) were chosen for further study, and we confirmed that miR-217 induced hypermethylation of the p16 and pRb promoters and influenced p16 and pRb expression though DNMT1. Our data indicated that the miR-217/DNMT1/p16/pRb axis contributed to HSF senescence. Finally, we demonstrated a negative correlation between miR-217 and DNMT1 expression during skin aging *in vivo*, implying that miR-217-dependent regulation of DNMT1 may occur *in vivo* and contribute to the pathogenesis of skin aging.

In summary, our study showed that miR-217 plays an important role in the HSF senescence through a mechanism involving the regulation of p16 and pRb methylation levels via targeting DNMT1. These findings raise the possibility that medical strategies aimed at protecting against the effects of miRNAs that promote skin aging, such as miR-217, could be effective in preventing aging. Adaptive regulation of miR-217 and DNMT1 merits further *in vivo* research in the field of skin aging.

## MATERIALS AND METHODS

### Skin tissues and cell culture

Skin tissues were collected from normal UV-unexposed areas surrounding the surgical sites from patients with benign dermatosis in the Department of Dermatology at the Xiangya Hospital of Central South University in Changsha of China. Skin tissues from 1–10-year-old and ~65-year-old patients were designated as “young skin tissues” and “old skin tissues,” respectively.

Primary normal HSFs were isolated from human skin by digestion with type-II collagenase (Sigma-Aldrich, USA) and cultured in Dulbecco's Modified Eagle's Medium (DMEM) with 20% FBS (Gibco, USA), as described previously [42]. Young-aged HSFs passaged 3–5 times were designated as “young HSFs”, while those passaged 20–25 were designated as “passage-aged HSFs”, and cells obtained from donors with varying ages were designated as “different-aged HSFs”.

This study was approved by the ethics committee of Central South University, and informed consent was obtained from all of the patients. The methods were carried out in accordance with the approved guidelines.

### RNA isolation and miRNA quantification

Total RNA was isolated from HSFs according to the manufacturer's instructions (Thermo Scientific). Single-stranded cDNA was synthesized using a kit from Thermo Scientific. Quantitative real-time PCR (qRT-PCR) was performed according to an miRNA protocol from Thermo Scientific using miR-217- or U6-specific primers (RiboBio Company, Guangzhou, China), inventoried real-time miRNA expression assays, and a RT-qPCR machine from Thermo Scientific. The comparative CT (ΔΔCT) method was used to calculate the real time quantitative (RQ) of miRNA expression, using U6 as a reference gene.

### Lentivirus and adenovirus transfection

HSFs (1 × 10^6^) in 6-well plates (Corning, USA) were washed twice in 1 mL of PBS, after which 2 mL antibiotic-free DMEM with 20% FBS was added. Two microliters of hsa-miR-217, hsa-miR-217 inhibitors, DNMT1-shRNA, a negative-control lentivirus, or a DNMT1 adenovirus (20 nM, GeneChem Company, China) were added to the culture medium, and the cells were incubated at 37°C for 48 h.

### Transfection of miRNA mimics and inhibitors

HSFs and 293T cells were transfected with an miR-217 mimics, an miR-217 inhibitors, or a scrambled miRNA control at a final concentration of 20 mM using Lipofectamine (Thermo Scientific), according to the manufacturer's instructions. The cells were subsequently incubated at 37°C for 48 h.

### Staining for senescence-associated β-galactosidase activity

HSFs were cultured as described above until they reached ~80% confluency and were then washed with PBS. The samples were fixed with 1 mL stationary liquid (β-Galactosidase Activity Staining Kit; Cell Signaling Technology Company, USA) per culture dish at room temperature for 15 min. After fixation, the samples were washed 3 times with PBS for 3 min/per wash and incubated at 37°C overnight in a sealed container (to avoid liquid evaporation), which was filled with freshly prepared staining solution (β-Galactosidase Activity Staining Kit). On the next day, staining was visualized under a microscope, and the aging rate of the cells was calculated.

### 3-(4, 5-Dimethylthiazol-2-yl)-2,5-diphenyltetrazolium bromide (MTT) assay

HSFs cultured as above were seeded in a 96-well culture dish at 5 × 10^3^ cells/well in 180 μL. Sterile PBS was added to well lining the edge of the dish. The culture dish was placed in an incubator at 37°C in a 5% CO_2_ air atmosphere. Twenty microliters of MTT stock solution (5 mg/mL MTT reagent diluted in PBS; Sigma-Aldrich, USA) was added to each well. The culture dish was further incubated for 4 h at 37°C and 5% CO_2_ in the dark. The supernatant was carefully removed without disturbing the sediment, and 150 μL dimethyl sulfoxide (Sigma-Aldrich, USA) was added to the wells to dissolve the purple formazan crystals. Absorbance of the samples was measured at 490 nm. All measurements were repeated 3 times to minimize deviations.

### Western blotting

In this experiment, western blot was used for detection of the expression of tubulin, DNMT1, p16, and pRb in fibroblasts. Initially, after the cells were washed with PBS, whole cell proteins were collected by treating the cells with a solution of 2%SDS (sigma, USA) sample buffer supplemented with protease inhibitors. And protein concertrations were detected by bicinchoninic acid method (BCA Protein Assay Kit, Thermo, USA) as the supplier instructed. Total protein of 50μg were loaded on 10% SDS-polyacrylamide gels and subjected to SDS-PAGE (SDS-PAGE Gel Parparation Kit, Beyotime, China). Protein bands were transferred to a polyvinylidene fluoride filter (PVDF, Pierce Chemical, USA), followed by block with 5% milk 5% fat-free milk in TBST buffer (100 mM NaCl, 10 mM Tris-HCl, pH 7.5, 0.1% Tween-20) for 1h at room temperature. Then the filter was incubated with special antibodies against tubulin, DNMT1, p16, and pRb (Abcam, Cambridge, UK) at 4°C overnight. Secondary antibodies marked with horseradish peroxidase (Santa cruz, USA) were used to incubate the filter for 1h at room temperature. Immunoreactive proteins were detected by protocols given in the ECL Plus kit (Beyotime, China), and the protein levels were quantitatively measured using the SuperSignal West Pico Chemiluminescent Substrate (Thermo Scientific, USA).

### Luciferase reporter assays

The 3′-UTR of the DNMT1 gene was amplified by PCR from genomic DNA and inserted into the pGL3 control vector (Promega, Madison, WI), using the XBA1 site immediately downstream of the stop codon of luciferase. The sequences of primers used to amplify the DNMT1-3′-UTR were: forward (5′-GGAGGAGGAAGCTGCTAAGG-3′) and reverse (5′-TTGGTTTATAGGAGAGATTTATTTG-3′) [43]. We also constructed the mutant reporter gene using the QuikChange Lightning Multi Site-Directed Mutagenesis Kit from Stratagene Corporation (USA). Each vector, along with 50 nM miR217, was transfected into HEK293 cells using the Lipofectamine 2000 reagent (Invitrogen), according to the manufacturer's instructions. Firefly and Renilla luciferase activities were measured consecutively using the Dual-Luciferase Reporter Assay System (Promega) at 48 h post-transfection.

### Detecting the promoter-methylation levels of senescence genes

DNA extraction and bisulfite conversion were performed using the EZ DNA Methylation-Lightning^TM^ Kit (Zymo Research, Irvine, CA, USA), according to the manufacturer's instructions. Bisulfite sequencing primers were designed for each gene using the online design tool, MethPrimer (http://www.urogene.org/methprimer/). High-throughput microfluidic PCR for target enrichment and next-generation bisulfite sequencing was performed, as described previously [44]. After MiSeq sequencing, the paired-end read data were de-multiplexed according to sample-specific barcodes with default parameters, using MiSeq Reporter software. The sequencing reads were mapped to each gene reference sequence using BiQ Analyzer software, version 3.0 with default parameters. The methylation status and level of each analyzed CpG-site in each gene were calculated using the BiQ Analyzer software. The methylation level of each gene was assigned by averaging the methylation level of all CpG sites in the gene for each sample.

### Statistical analysis

All data were expressed as the mean ± standard error of the mean from at least 3 independent experiments. Statistical significance was tested with repeated analysis of variance (ANOVA) using a least-significant difference post-hoc test or ANOVA for multiple comparisons (SPSS software, version 19.0). Linear regression analysis (R^2^) was used for correlation analysis. Differences were considered significant at p < 0.05.

## SUPPLEMENTARY MATERIALS FIGURES AND TABLES


