# Supplementary material for: Microrna-217 modulates human skin fibroblast senescence by directly targeting DNA methyltransferase 1
Source: Oncotarget. 2017 Mar 23;8(20):33475–86. doi: 10.18632/oncotarget.16509 (PMC5464883; doi:10.18632/oncotarget.16509)
Supplement: Supplementary file 1 [file oncotarget-08-33475-s001.pdf]

Microrna-217 modulates human skin fibroblast senescence by directly targeting DNA methyltransferase 1

SUPPLEMENTARY MATERIALS

SUPPLEMENTARY FIGURE AND TABLE

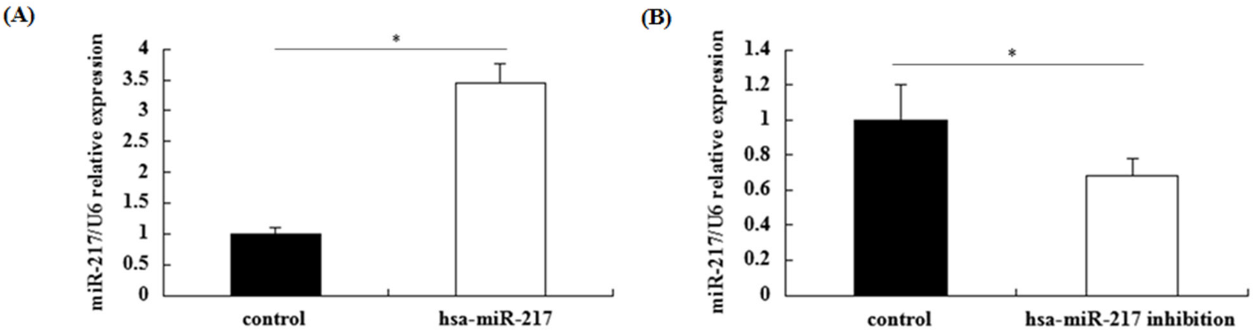

Ssupplementary Figure 1: miR-217 levels in HSFs transduced with the hsa-miR-217 or hsa-miR-217 inhibitors lentivirus. **(A)** miR-217 levels were detected by RT-qPCR. miR-217 levels significantly increased after transduction with the hsa-miR-217 lentivirus in young HSFs (n = 3, *$p < 0.05$). **(B)** miR-217 levels were detected by RT-qPCR. miR-217 levels significantly decreased after transduction with the hsa-miR-217 inhibitors lentivirus in passage-aged HSFs. (n = 3, *$p < 0.05$)

Supplementary Table 1: The list of 24 senescent-associated genes

The abbreviation of genes	Genes' full names
pRb	Phosphorylated retinoblastoma
ATM	Ataxia telangiectasia mutated
NF-κB1	Nuclear factor of kappa light polypeptide gene enhancer in B-cells 1
Sirt1	Sirtuin 1
NANOG	Nanog homeobox
SP1	Sp1 transcription factor
SOX2	Sex determining region Y-box 2
VDR	1,25-dihydroxyvitamin D3 receptor
ZEB1	Zinc finger E-box binding homeobox 1
ZEB2	Zinc finger E-box binding homeobox 2
PTEN	Phosphatase and tensin homolog
Foxd3	Forkhead box D
Dnmt3a	DNA (cytosine-5-)-methyltransferase 3 alpha
Dnmt3b	DNA (cytosine-5-)-methyltransferase 3 beta
p53	P53 tumor suppressor
p21	CDKN1A
p16	CDKN2A
LEF1	Lymphoid enhancer-binding factor 1
UTF1	Undifferentiated embryonic cell transcription factor 1
TERT	Telomerase reverse transcriptase
SFRP2	Secreted frizzled-related protein 2
KIT	Kit oncogene
GRB7	Growth factor receptor-bound protein 7
CTNNB1	Cadherin-associated protein, beta 1
